# Overcoming data scarcity in life-threatening arrhythmia detection through transfer learning

**DOI:** 10.1038/s43856-025-00982-9

**Published:** 2025-07-01

**Authors:** Giuliana Monachino, Beatrice Zanchi, Michael Wand, Giulio Conte, Athina Tzovara, Francesca Dalia Faraci

**Affiliations:** 1 Institute of Digital Technologies for Personalized Healthcare - MeDiTech, Department of Innovative Technologies, University of Applied Sciences and Arts of Southern Switzerland, Lugano, Switzerland; 2 Institute of Informatics, University of Bern, Bern, Switzerland; 3 Department of Quantitative Biomedicine, University of Zurich, Zurich, Switzerland; 4 Dalle Molle Institute for Artificial Intelligence, USI-SUPSI, Lugano, Switzerland; 5 Cardiology Department, Cardiocentro Ticino Institute, Ente Ospedaliero Cantonale, Lugano, Switzerland; 6 Faculty of Biomedical Sciences, Universitá della Svizzera Italiana, Lugano, Switzerland; 7 Sleep Wake Epilepsy Center ∣ NeuroTec, Department of Neurology, Inselspital, Bern University Hospital, University of Bern, Bern, Switzerland

**Keywords:** Arrhythmias, Diagnosis

## Abstract

**Background:**

Life-threatening arrhythmias (LTAs) are a leading cause of death worldwide. Enhancing LTA detection in wearable monitoring systems is of great importance. One of the main challenges in building robust LTA detection algorithms is the limited availability of labeled LTA data.

**Methods:**

We introduce an effective deep-learning algorithm for detecting LTAs from single-lead ECGs in out-of-hospital cardiac arrest applications. We address the data-scarcity issue by applying a transfer learning approach. The deep-learning model is pre-trained on a massive dataset (72’952 recordings) for rhythm classification and then fine-tuned on the target dataset with LTA events (102 recordings).

**Results:**

Our model achieves a sensitivity of 92.68% and a specificity of 99.48%, with a granularity of 1.28 seconds, in detecting LTAs. Additionally, a confidence estimation procedure is introduced to enable emergency service pre-alerts in case of low-confidence detections.

**Conclusions:**

Our transfer learning based approach has the potential to significantly mitigate the impact of data scarcity, advancing LTA detection in wearable monitoring systems, and supporting rapid, life-saving interventions in out-of-hospital cardiac arrest emergencies.

## Plain Language Summary

Life-threatening arrhythmias (LTAs) are critical cardiac conditions that can lead to sudden cardiac death, frequently occurring outside of clinical settings. Early detection of these events using wearable devices has the potential to significantly increase survival rates. However, developing reliable automated detection systems remains challenging due to the limited data availability on these rare events. In this study, we propose a deep-learning model that leverages transfer learning: it initially learns from a large dataset of general cardiac signals and subsequently adapts to a smaller dataset specific to LTA cases. The model demonstrates high accuracy in identifying LTAs and provides a confidence measure for each detection. This approach may facilitate timely emergency responses and improve outcomes in real-world scenarios involving sudden cardiac arrest.

## Abstract (web summary)

Monachino et al. investigate how to overcome the challenge of data scarcity in life-threatening arrhythmias (LTA) detection. They propose a transfer learning-based deep learning model that detects LTAs from single-lead ECGs with 92.68% sensitivity and 99.48% specificity and includes a confidence score to better support emergency response.

## Introduction

Life-threatening arrhythmias (LTAs), such as ventricular fibrillation (VF) and ventricular tachycardia (VT), are a critical public health concern. These conditions disrupt the heart’s normal electrical activity, resulting in irregular and often dangerously rapid heartbeats that hinder the heart’s ability to pump blood efficiently. This can quickly lead to sudden cardiac arrest or sudden cardiac death.

One of the critical contexts where LTAs become especially dangerous is when they occur unexpectedly outside of the hospital setting. In this case, they can lead to the so-called out-of-hospital cardiac arrest (OHCA): the heart unexpectedly stops beating, leading to an immediate cessation of blood flow to vital organs. Prompt recognition and intervention are crucial to survival, as every minute of delay in defibrillation considerably decreases the chances of a successful resuscitation^1,2^.

Sudden cardiac arrest, and hence OHCA, is more likely to occur in people suffering from inherited arrhythmogenic diseases (IADs)^3^ such as long QT syndrome, Brugada syndrome, and early repolarization syndrome. IADs, indeed, considerably increase the likelihood of LTA development, and hence the risk of sudden cardiac arrest.

In the last decades, artificial intelligence (AI) has emerged as a powerful tool in the field of cardiology, offering promising advancements and support for cardiologists in diagnosis, treatment, risk stratification, and early prediction of cardiac diseases^4,5^. AI algorithms, exploiting machine learning (ML) and deep learning (DL) techniques, have demonstrated the ability to analyze vast amounts of electrocardiogram (ECG) data with high accuracy, identifying subtle patterns and anomalies that may elude human detection.

In the context of LTAs and OHCA, the potential use of wearable sensors adds another layer of innovation in the early detection and management of this dangerous condition. These sensors, often integrated into smartwatches, belts, or sensorized vests, combined with AI, may allow to continuously monitor heart rhythm and other vital signs and detect arrhythmic events earlier, triggering alerts for both patients and healthcare providers, allowing for prompt medical intervention^6–8^.

This study has been conducted in the framework of the European project CMIPA (E115814) focused on people suffering from IADs. It aimed to provide: continuous monitoring through a sensorized vest (i.e., Healer TeleHealth System produced by L.I.F.E. Italia S.r.l.), real-time detection of LTAs, and automatic alert to emergency services to ensure prompt intervention when needed. This study represents a preliminary step: we developed a robust DL-based algorithm, able to detect the presence of LTAs from a single-lead ECG signal in real-time. The ultimate objective is to integrate the proposed algorithm into the system, feeding it with data from the wearable sensors.

To the best of our knowledge, this is the first algorithm developed for this specific application. However, our objective is comparable to a similar, moderately explored task: the classification of shockable (Sh) vs non-shockable (NSh) rhythms. Shockable rhythms, indeed, include VT and VF. This binary classification is the core of shock advice algorithms (SAA), which are integrated into automated external defibrillators to control the release of the electric shock, especially during OHCA. Another pertinent application is the identification of LTAs in implantable cardioverter-defibrillators^9,10^. However, the analytical approach differs substantially as it relies on intracardiac electrogram instead of surface ECG.

We report the relevant literature related to Sh vs NSh rhythms classification only for having a reference, since this is the closest task, but the task addressed in this work is different. We aim to identify any LTA regardless of whether they are shockable and/or lead to cardiac arrest. For safety and device design reasons, this differentiation is left to the expert rescuers automatically called by the system in the event of an alarm.

As reported by Nguyen et al.^11^, SAA can be categorized into threshold-based, intelligent ML-based, and intelligent DL-based approaches. The first ones are the simplest but usually reach a relatively low sensitivity, failing to meet the American Heart Association (AHA) requirements^12^ for SAA performance (at least 90% sensitivity and 95% specificity). AI-based algorithms, instead, reach higher performance, complying with AHA guidelines. Most of the works proposed in the literature for the three categories can be found in the review presented by Nguyen et al.^11^. We report here the most representative candidates for the two AI-based groups according to the review. The best performing for the ML group is the algorithm proposed in the review itself^11^. The authors adopt a support vector machine on features extracted from an 8s ECG signal augmented with modified variational mode decomposition, reaching 98.2%–99.8% of sensitivity-specificity. Instead, for the DL group, Nguyen et al.^13^ exploit a convolutional neural network (CNN) to extract features from an 8s ECG signal augmented with modified variational mode decomposition and a boosting approach for the classification, resulting in 97.1%–99.4% of sensitivity-specificity. It is relevant to highlight that all the algorithms reported in the review^11^ are trained with a quite limited number of ECG recordings (usually around one hundred), which are extracted from the only available open-access^14–17^ or on-request^17,18^ databases, or from private databases^19,20^ containing episodes of LTAs. This can lead to a high risk of overfitting and low generalizability over new, unseen data, due to the low heterogeneity and low representativeness inherent in the limited number of subjects.

It is worth mentioning the work of Dahal et al.^21^, since the authors combined a generative adversarial network with a deep neural network to synthesize ECG recordings. The goal is to increase the training set size and boost the algorithm’s performance. They achieved 96.76%–99.56% of sensitivity-specificity. The work presented by Shen et al.^22^, instead, is the one with the largest number of subjects exploited for SAA. Indeed, by combining public and private datasets, they managed to exploit a total of 26’464 ECG recordings. With a 6-layer CNN, they reached a sensitivity of 98% and a specificity of 100%. However, these results have been obtained by excluding the conflict ECGs, i.e., the segments for which the 3 scorers had not unanimously assigned the Sh/NSh label. Later, they also tested the algorithm on the conflict ECGs, obtaining an agreement of 84.4% with the 2/3 majority classification of the scorers. This highlights how much the problem related to noisy or imprecise labels affects the final performance of the algorithm. Other works^20,21,23^ faced this issue by relabeling the exploited public datasets: the original labels were revised by two experienced biomedical engineers. Moreover, further works^11,13,19–21,23,24^ applied a screening procedure to exclude ECG excerpts with noise, asystole, transition rhythm, and intermediate rhythms such as slow VT or low amplitude VF, maybe due to recommendations related to the SAA development.

Despite their impressive performance, the works in the literature present some non-negligible limitations. The most frequent one is the risk of limited generalizability due to the low heterogeneity and size of the training datasets. Moreover, as mentioned above, several works exclude the most critical cases from the study (e.g., noisy ECGs or labels, transition rhythms, etc.), leading to less realistic evaluations. Finally, in all the cases, we observed a lack of validation of the proposed algorithms on out-of-domain data, which would allow for a fairer performance evaluation. However, the latter is hard to address due to the unavailability of a benchmarking dataset.

In this work, our main goal is to overcome the main limitation related to this field of application: the low availability of labeled ECG recordings containing LTA events for training a robust model. To accomplish this, we propose to take advantage of the high availability of ECGs associated with other tasks by exploiting a transfer learning approach with task shifting. Thus, our main research question is whether it is possible to extract valuable knowledge from a massive amount of ECG data and effectively transfer it to the target task (i.e., LTA detection), despite differences in characteristics such as tasks and labels. First, we pre-train a DL model on a temporary support task to extract information from the pre-training datasets. Then, we fine-tune the pre-trained model on the target task, exploiting the few ECG datasets available with LTA events. In the present study, we do not exclude any segment and exploit the original labels provided in the openly available datasets.

The following experiments are conducted: (i) Comparison of the proposed transfer learning approach with the standard approach of training the model from scratch. (ii) Search for the optimal configuration for fine-tuning the model on the target task. (iii) Examination of the possibility of reducing the model size in the fine-tuning phase to obtain a lighter model without losing classification power. (iv) Exploration of how to evaluate the algorithm’s prediction confidence and analysis of both stationary and transitory rhythm phases.

Our main contributions can be summarized as follows. (1) The LTAs-ECG data scarcity problem can be overcome by extracting knowledge from data belonging to another domain without LTA events. The presented transfer learning approach, combined with an optimized version of a state-of-the-art CNN capable of achieving cardiologist-level results^25^, improves model generalizability and performance. This approach also allows the exploitation of a lighter version of the CNN for the downstream task while keeping the performance almost unchanged. (2) A robust DL LTAs identification algorithm is developed: it is capable of detecting LTAs from single-lead ECGs, with a granularity of 1.28s and with high performances (92.68% sensitivity and 99.48% specificity). (3) A total of 72’952 recordings from 71’240 different subjects are included, resulting in 83’949 9s-ECG sequences, the biggest dataset employed in similar studies. (4) A confidence evaluation approach is proposed to pre-alert the emergency services in case of LTAs, reducing the negative consequences of false positives and false negatives.

## Methods

### Datasets and preprocessing

For this study, we constructed two sets of ECG data: the pre-training dataset (PT) and the fine-tuning LTA dataset (FT-LTA). Table 1 reports the final number of subjects, recordings, and segments in each dataset, divided by subset (train, validation, and test).

**Table 1:** Summary of FT-LTA and PT datasets characteristics

	Train			Validation			Test		
	Sbj	Rec	Segm	Sbj	Rec	Segm	Sbj	Rec	Segm
PT	43144	45497	318479	10782	11064	77448	16212	16289	114023
FT-LTA	67	67	51898	15	15	8218	20	20	17577
- LTA	–	–	6574	–	–	945	–	–	1470
- Other	–	–	45324	–	–	7273	–	–	16107

Number of subjects, recordings, and segments are reported for each subset (train, test, validation) of the PT and FT-LTA datasets. For the two classes of FT-LTA (LTA and Other), the number of segments is also reported.

#### PT dataset

The PT dataset aggregates four open-access datasets from *PhysioNet/Computing in Cardiology Challenge 2021*^17,26–28^: Ningbo^29^, Chapman-Shaoxing^30^, PTB-XL^31^, and The Georgia 12-lead ECG Challenge Database.

Chapman-Shaoxing dataset study was approved by the institutional review board (IRB) of Shaoxing People’s Hospital, which granted the waiver application to obtain informed consent, and allowed the data to be shared publicly after de-identification. Ningbo dataset study was approved by the IRB of the Ningbo First Hospital of Zhejiang University, which granted the waiver of the requirement to obtain informed consent. The PTB-XL dataset was collected and curated by the Physikalisch-Technische Bundesanstalt and the Institutional Ethics Committee approved the publication of the anonymous data in an open-access database (PTB-2020-1). All these datasets contain fully anonymized, retrospective data, and no additional IRB approval was required for the current secondary analysis.

All of them contain 12-lead ECG recordings, sampled at a frequency of 500 Hz. Ningbo Database originally includes 34’905 recordings (age range: 0–89 y, average: 57.7 ± 20.4 y, percentage of females: 43%), Chapman-Shaoxing Database 10’646 recordings (age range: 4–89 y, average: 60.1 ± 17 y, percentage of females: 44%), Georgia Database 10’344 recordings (age range: 14–89 y, average: 60.5 ± 15.4 y, percentage of females: 46%), and PTB-XL Database 21’837 recordings from 18885 subjects (age range: 2–89 y, average: 59.5 ± 16.8 y, percentage of females: 48%). Exact age of subjects older than 89 is not available for compliance with HIPAA standards. All these datasets contain 10-second ECG signals, except for the Georgia Database, whose instances last 5 (less than 2%) or 10 s. Each ECG is provided with a set of labels describing cardiac diagnosis, morphology, and/or rhythm, assigned to the whole recording period and encoded through the SNOMED-CT standard.

From these four datasets, we included the recordings containing at least one rhythm label in the PT dataset and extracted only lead II. As regards the Georgia Database, we excluded the ECGs lasting 5 s. Afterward, ECG recordings associated with the Noise label or underrepresented labels (around 3% of the remaining recordings) have been removed. A total of 72850 ECG signals from 70138 subjects associated with 14 different combinations of rhythm labels (multiple labels are associated with the same recording) make up the final dataset.

All the ECG signals have been resampled at 200 Hz. Each 10s recording has been truncated to obtain a sequence made by 7 256-sample segments (about 8.96s), to ensure compatibility with the architecture used during the pre-training phase. From the set of labels associated with each recording, we selected only the rhythm labels, resulting in one or more rhythms assigned to each sequence. The signals have been filtered with a Butterworth 4th order filter with cutoff frequencies of 0.5 and 40 Hz.

The sequences in the PT dataset have been split on a subject basis and by exploiting a multi-label stratified strategy. 20% of the dataset has been kept unused for the test. The remaining 80% has been split into 80% for training and 20% for validation. Then, the data were standardized based on the mean and standard deviation computed on the training set.

The resulting number of subjects, recordings, and sequences for each source dataset of PT are reported in Supplementary Table 1, while the PT labels distribution can be found in Supplementary Table 2. It is worth noting that LTAs (e.g., ventricular tachycardia, ventricular fibrillation) are not present in PT recordings.

#### FT-LTA dataset

The FT-LTA dataset groups three publicly available datasets widely used in the literature for the Sh vs NSh rhythm classification task: MIT-BIH arrhythmia database (MITDB)^16,17,32^, MIT-BIH malignant ventricular ectopy database (VFDB)^14,17,33^, and Creighton University ventricular tachyarrhythmia database (CUDB)^15,17,34^.

All these datasets contain publicly accessible, anonymized, retrospective data, hence no additional IRB approval was required for the current secondary analysis.

MITDB contains 48 30-minute ECG recordings, sampled at 360 Hz, from 47 subjects (46% females) aged between 23 and 89 (average 63.7 ±17.7 y) studied by the Beth Israel Hospital Arrhythmia Laboratory between 1975 and 1979. VFDB contains 22 30-min ECG recordings, sampled at 250 Hz, of subjects who experienced episodes of sustained ventricular tachycardia, ventricular flutter, and ventricular fibrillation. CUDB contains 35 8.5-min single-lead ECG recordings, sampled at 250 Hz, of subjects who experienced episodes of ventricular fibrillation. Demographic (age and sex) information for VFDB and CUDB datasets are not abailable. For all three datasets, rhythm annotations were provided, with the time reference of each rhythm change. For MITDB, also beat labels were available.

To build the FT-LTA dataset, we extracted lead II and selected the labels describing cardiac rhythm changes. The FT-LTA dataset includes a total of 104 ECG recordings from subjects who have experienced LTAs.

All the recordings have been down-sampled to 200 Hz and converted to uV for consistency with the pre-training data. Each recording has been split into non-overlapping sequences of 7 256-sample segments. The rhythm labels have been divided into two classes: LTA, including ventricular tachycardia (VT), ventricular fibrillation (VF), ventricular flutter (VFL), and polymorphic ventricular tachycardia (PVT), and Other, including all the other rhythms. Then a unique class (LTA or Other) was associated with each 256-sample segment, obtaining 7 labels per sequence. The most frequent class has been assigned in case of multiple rhythms. The segments labeled with Noise have been removed since a clear rhythm could not be assigned.

Each sequence has been pre-processed following the procedure proposed by Amann et al.^35^ and also exploited by other works in the field^20,21,23^. It consists of four steps: mean subtraction, a five-order moving average filter, a high-pass filter at 1 Hz for drift suppression, and a low-pass Butterworth filter at 30 Hz. The 1-30 Hz is a typical monitor bandwidth used in AEDs to distinguish shockable vs non-shockable rhythms.

Each of the three datasets has been split on a subject basis into train, validation, and test sets. 20% of the dataset has been kept unused for the test, while the remaining 80% has been split into 80% for training and 20% for validation. Finally, the whole dataset was standardized based on the mean and standard deviation calculated on the training set.

Table 1 reports the label distribution in each subset of the FT-LTA dataset, while Supplementary Table 3 also reports the label distribution for each source dataset.

### ECGnet architecture

The architectures exploited in this study are adaptations of the one proposed by Hannun et al.^25^ for arrhythmia classification. For simplicity, in this work, we will name it ECGnet.

#### ECGnet

ECGnet takes as input a single-lead raw ECG signal sampled at 200 Hz and provides as output 1 of the 12 rhythm classes every 256 samples (1.28 seconds). It is a CNN with shortcut connections, composed of 16 residual blocks. Each block includes two sets of batch normalization (BN), rectified linear (ReLU) activation, and convolutional layers. The second convolutional layer is preceded by a dropout layer with a probability of 0.2. The number of filters in the convolutional layers doubles every fourth residual block, starting at 32. A subsampling of a factor of 2 is applied to each alternate residual block’s inputs. The last block consists of a BN, ReLU, and a fully connected (FC) layer with softmax activation. The last FC layer is applied to every temporal slice of its input independently, sharing the same weights. Thanks to this structure, ECGnet can be exploited for ECG signals of any length, as long as they contain a multiple of 256 samples (sampled at 200Hz).

For further details about the original architecture, we refer the reader to the reference work^25^.

#### Adaptations: ECGnet-v0, ECGnet-v1, ECGnet-v1-M, ECGnet-v1-S

In our work, we adapted ECGnet for two specific tasks.

The first pre-training task was a multi-class classification. ECG recordings are associated with multiple ground truth labels, which are assigned to the entire duration of the recording. Importantly, these labels lacked specific temporal references, meaning that no start or end time points were provided for individual events within the recording. To adapt ECGnet to this task, we modified the last layers, obtaining ECGnet-v0. We first substituted softmax with a sigmoid activation function to obtain a probability for each of the 10 possible rhythm classes. We also added a global max pooling layer to output a single prediction (set of labels) for the whole sequence, while keeping the TimeDistributed layer to preserve input length independence. The second task was the LTA detection task. We set the number of target classes equal to two for the binary classification LTA vs Other. We refer to this network as ECGnet-v1. A graphical representation of ECGnet-v0 and ECGnet-v1 is provided in Supplementary Fig. 1.

In the original work, the input was raw data. Instead, we input to the network ECG signals pre-processed to reduce noise. For the training phase, we fixed the input length to 7 segments of 1.28s, i.e., to an 8.96s sequence. Hence, we truncated and/or segmented the recordings accordingly. In this way, we removed the zero-padding exploited in the original work to equalize the size of all inputs, thereby eliminating a possible source of bias. We also introduced data shuffling before batch creation. As a loss function, we used the binary cross-entropy loss for both ECGnet-v0 and ECGnet-v1.

As regards the hyperparameters and optimization algorithm, we kept the ones optimized in the reference work^25^. Early stopping based on validation loss with a patience of 30 epochs and a maximum number of training epochs equal to 500 has been exploited. We increased the batch size to 512 for the pre-training.

For the network lightning experiment (see Experiment section), we built two reduced versions of ECGnet-v1: ECGnet-v1-M and ECGnet-v1-S. Specifically, we reduced the number of residual blocks to 3/4 and 1/2 of the original size, respectively. To preserve consistency in input and output dimensions, we doubled the number of filters in convolutional layers every third or second residual block, respectively, and adapted the sub-sampling accordingly, as represented in Supplementary Fig. 2.

### Transfer learning

Transfer learning can be defined with the following statements:

“Transfer learning and domain adaptation refer to the situation where what has been learned in one setting (e.g., distribution *P*_1_) is exploited to improve generalization in another setting (say, distribution *P*_2_)”^36^;

“Given a source domain *DS* and learning task *TS*, a target domain *DT* and learning task *TT*, transfer learning aims to help improve the learning of the target predictive function *fT*( ⋅ ) in *DT* using the knowledge in *DS* and *TS*, where *DS* ≠ *DT* and *TS* ≠ *TT* ”^37^.

This approach is particularly beneficial when the target task has limited labeled data. The key idea is to transfer the representations learned from a related task with extensive source data to the target task, allowing the model to generalize better and learn more efficiently. This process can involve a second phase of fine-tuning, where the pre-trained model is further trained on the target data.

In this work, we exploited transfer learning to address the problem of the low availability of labeled data for the life-threatening arrhythmia detection task. We had both a domain shift (*DS* ≠ *DT*), due to the differences between source and target datasets (different recording settings and hardware, subject demographic and clinical characteristics, ECG properties, etc...), and a task shift (*TS* ≠ *TT*), with arrhythmia classification as the pre-train task and life-threatening arrhythmia detection as the target task. Thus, we also performed a fine-tuning step on the target dataset, starting from the pre-trained model. To avoid the so-called catastrophic forgetting, we also partially froze the neural network during the fine-tuning.

### Confidence estimation

We introduced a method to estimate the model prediction’s confidence, which is based on the ECGnet property to provide an output every 1.28 seconds. This approach is represented graphically in Fig. 2A.

The confidence is computed as follows. Consider a window with a length of the input size (8.96s, 1 sequence) that slides on an ECG signal with a 1.28-second step (1 segment). Applying ECGnet-v1 to each window will result in 7 predictions for each segment. The final label (LTA vs Other) is assigned with majority voting, and the confidence value is obtained by dividing the number of segments predicting the final label by 7 (the total number of segments).

### Experiments

#### (i) Training from scratch vs transfer learning with task shift

The main experiment aims to assess whether exploiting a transfer learning technique with domain and task shift could improve the ECGnet performance in the LTA detection task. In other words, we aim to evaluate whether exploiting the knowledge learned from a massive amount of data belonging to another domain (different classification tasks and other ECG rhythms) could be beneficial in solving the target downstream task. For this purpose, we considered the training from scratch as our baseline, to be compared with the application of transfer learning with task shift. To perform a fair comparison, both models have been tested on the same set of data, i.e., the FT-LTA test set.

To build our baseline (ECGnet-v1 TS), we exploited the ECGnet-v1 to solve a binary classification task (LTAs vs Other). The model was trained from scratch using the FT-LTA training set. To address the problem of the unbalanced FT-LTA dataset, we balanced each training batch by over-sampling the less represented class (LTA) three times and under-sampling the other one (Other) to reach the same number of samples in both classes. We selected the under-over-sampling approach after comparing it with another typical approach (weighted cross-entropy loss) and a baseline (without data balancing) and obtaining the best performance. For completeness, the results obtained with the two other approaches are reported in Supplementary Tables 4, 5.

To train the second model (ECGnet-v1 TL), instead, we applied the transfer learning technique that involves two steps: pre-training and fine-tuning.

We first pre-trained ECGnet-v0 to solve a multi-label arrhythmia classification task. The adoption of ECGnet-v0 was necessary to adapt the final part of the architecture to the multi-label multi-class classification task. The model was pre-trained using the PT training set. The PT test set, instead, has been exploited to ensure that the model has been trained well enough.

In the second phase, as for the baseline, we adopted ECGnet-v1 to solve the binary classification task. In this case, the model has been initialized with the pre-trained model’s weights, extracted from the common part of ECGnet-v0 and ECGnet-v1 (i.e., all except the last two layers). The initialized ECGnet-v1 was then fine-tuned on the FT-LTA training set, as for the baseline.

#### (ii) Optimization of the freezing configuration

In this experiment, we partially froze the network during the fine-tuning phase. The aim was to take advantage of more of the information learned during pre-training to reduce the risk of overfitting and increase model generalizability. To find a compromise between the exploitation of pre-training information (more layers frozen) and the adaptation to the downstream task (fewer layers frozen), in this experiment, we optimized the freezing configuration (ECGnet-v1 TL-opt). We performed the fine-tuning multiple times by always initializing the whole network with the pre-trained weights, but gradually increasing the number of frozen residual blocks. We then compared the performance on the FT-LTA validation set to select the optimal number of residual blocks to be frozen. Finally, we compared the performance obtained on the FT-LTA test set, adopting the best configuration (ECGnet-v1 TL-opt), with the baseline of the previous experiment (ECGnet-v1 TS).

#### (iii) Network lightning

We also tried to reduce the ECGnet size, aiming to evaluate the possibility of having a lighter network to reduce computational complexity. Given the small size of the FT-LTA dataset, we expected that reducing the network size and hence lowering its complexity could still bring good performance and may reduce the risk of overfitting on the dataset. Specifically, we built two reduced-size ECGnet architectures (ECGnet-v1-M and ECGnet-v1-S). We trained and tested the two networks employing the transfer learning protocol (with the optimized number of frozen layers). We considered our ECGnet-v1 as the baseline (here named ECGnet-v1-L) to be compared with the other two reduced networks (ECGnet-v1-M and ECGnet-v1-S). We tested the three models on the FT-LTA test set.

#### (iv) Confidence estimation

In this last experiment, we tested the proposed confidence estimator, evaluating its association with the capability of the model to give a correct or wrong prediction. We first identified true/false positive/negative segments in the FT-LTA test set, and then we analyzed the distribution of the confidence values in each group.

We investigated the performance and confidence of the model in stationary and transitory phases to verify the hypothesis that most of the low-confidence or misclassified segments are located in correspondence to the transition between one type of rhythm and another. We defined stationary and transitory phases based on the ground truth. In transitory phases, we included the three segments before and after each point of change from one type of rhythm to another (LTA and Other). All the other segments have been considered as stationary. For each of the two phases, we calculated and compared the model performance and its confidence.

### Evaluation

To evaluate the performance on the target task, we exploited the following binary classification metrics: sensitivity, specificity, BER, accuracy, Cohen’s k, weighted F1 score (wF1), and macro F1 score (MF1). MF1 is the average of the per-class F1 values, while wF1 is the average weighted by the number of test samples in each class. The BER is defined as 1-BAC, where BAC is the balanced accuracy that is the mean between sensitivity and specificity.

When in doubt, we based our models’ comparison primarily on the MF1 since it takes into account both false positives and false negatives and considers the performance in both classes without favoring either of them based on the number of available samples.

### Statistics and reproducibility

To fairly compare the performance of different models, we exploited the one-sided Mann-Whitney U test, fixing a p-value of 0.05. It was performed, for all the aforementioned metrics, by considering the set of 10 predictions obtained with the 10 trained models for each experiment. This test has been performed using the Python SciPy library (scipy.stats.mannwhitneyu v1.11.2).

We also estimated the effect size through the rank-biserial correlation coefficient *r*, calculated as $r=1-\frac{2U}{{n}_{1}\cdot {n}_{2}}$, where U is the test statistic and *n*_1_ = *n*_2_ = *n* is the sample sizes, equal to 10.

### Reporting summary

Further information on research design is available in the Nature Portfolio Reporting Summary linked to this article.

## Results

### Training from scratch vs transfer learning with task shift

In the main experiment, we compared the application of the transfer learning approach with task shift (ECGnet-v1 TL) with our baseline, i.e., the standard training from scratch (ECGnet-v1 TS). The performance of ECGnet-v1 TS and ECGnet-v1 TL computed on the target FT-LTA test set is reported in the upper part of Table 2.

**Table 2:** Performance comparison for the experiments (i) and (ii)

Model	Se	Sp	BER	Acc	k	wF1	MF1
ECGnet-v1 TS	85.81 (6.86)	98.88 (1.11)	7.65 (3.31)	97.79 (1.02)	85.59 (5.98)	97.79 (0.96)	92.79 (3.00)
ECGnet-v1 TL	90.33 (4.03)*	99.02 (0.89)	5.33 (1.85)*	98.29 (0.73)	89.03 (4.21)	98.30 (0.69)	94.51 (2.11)
ECGnet-v1 TL opt	92.68 (3.35)*	99.48 (0.36)*	3.92 (1.57)*	98.92 (0.28)*	92.87 (1.84)*	98.91 (0.28)*	96.44 (0.92)*

Performance of ECGnet-v1 TS (training from scratch), ECGnet-v1 TL (transfer learning), and ECGnet-v1 TL-opt (transfer learning with optimized configuration) computed on the FT-LTA test set. Metrics are reported as mean (std) over the 10 models trained with different random initializations. The best results are marked in bold.

^*^ Significantly higher (lower in case of BER) than the baseline (ECGnet-v1 TS), according to Mann–Whitney U test (*p* < 0.05).

ECGnet-v1 TL shows a significant improvement (*p* < 0.05) with respect to the baseline in terms of sensitivity (+4.52%, *p* = 0.0481,  *r* = 0.45) and BER (−2.32, *p* = 0.0226,  *r* = −0.54). For all the other reported metrics, the Mann–Whitney U test shows that ECGnet-v1 TL is not inferior to the baseline. This implies that the adoption of a transfer learning approach, in this setting, is beneficial to solve the target task, especially to improve the model’s ability to detect the low-represented class (LTAs).

### Optimization of the freezing configuration

**Fig. 1:**
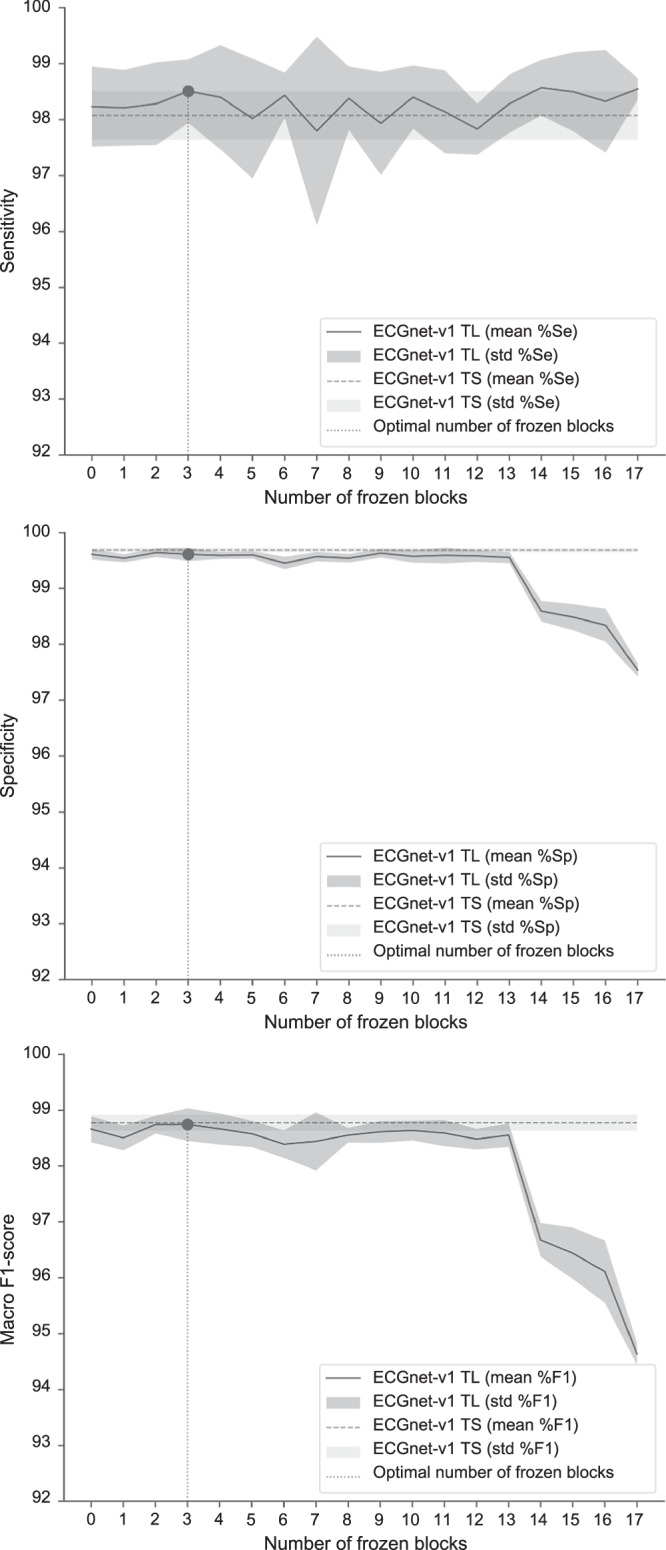
Sensitivity, specificity and macro F1-score of ECGnet-v1 TL on the FT-LTA validation set varying the freezing configuration. The x-axis reports the number of frozen blocks during the fine-tuning phase (from no frozen blocks to all but the last frozen block). The lines and the shadows represent, respectively, the means and the standard deviations across the *n* = 10 runs.

We also optimized the fine-tuning by partially freezing the pre-trained network during this phase. We identified the optimal number of layers to be frozen by testing different configurations on the FT-LTA validation set. We chose the optimal value relying on the macro-F1 score, due to the comprehensive nature of this metric. The results of the different configurations, in terms of sensitivity, specificity, and macro f1-score, are presented in Fig. 1. These metrics are reported in Supplementary Fig. 3, also for the FT-LTA test set, for comparison with the validation set. The performance obtained on the FT-LTA test set with the optimal configuration (ECGnet-v1 TL-opt) are reported in Table 2 and compared with the baseline and the ECGnet-v1 TL (without layer freezing).

The best configuration (on the FT-LTA validation set) turned out to be the one with only the first three residual blocks frozen during the fine-tuning phase: a sensitivity of 92.68% and a specificity of 99.48% is obtained. This means that the model takes advantage mainly of the pre-trained weights of the first layers, which extract the higher-level features. This matches the expectations, due to the low similarity between the pre-training and target task/data. Figure 1 shows how sensitivity remains quite stable, varying the number of frozen layers. Macro F1-score and specificity, instead, follow a different trend: they are highly stable as long as the number of frozen blocks does not exceed 13, while dramatically decreasing as this number further increases. This highlights the need for a fine-tuning phase, which adapts the model to the target task by acting on the unfrozen layers. Especially in this case, it reduced the number of false positives compared to directly using the pre-trained model for the target task.

The performance obtained in the FT-LTA test set with the optimal model reveals a clear superiority of the ECGnet-v1 TL-opt model with respect to the baseline, which is statistically significant (*p* < 0.05) for all the reported metrics. It results in an increase of sensitivity, specificity, and macro F1-score of 6.97% (*p* = 0.0009,  *r* = 0.83), 0.6% (*p* = 0.0378,  *r* = 0.48), and 3.65% (*p* = 0.0006,  *r* = 0.86), respectively. This further confirms that transfer learning is a winning approach for the extraction of useful information during the pre-training phase, even if both the target dataset and task differ quite from the ones exploited for the pre-training phase. Moreover, it highlights the importance of partially freezing the pre-trained layers to avoid catastrophic forgetting and take more advantage of the previously learned information. This is further supported by sensitivity, specificity, and macro F1-score’s trend upon variation of the number of frozen blocks, reported in Supplementary Fig. 3. These curves present a performance decrease also in the first part, highlighting the importance of the knowledge learned by the first blocks during the pre-training phase, which enhances the model’s generalizability.

### Network lightning

In this experiment, we evaluated the trade-off between the size of ECGnet-v1 and its classification power. Table 3 reports the performance obtained on the FT-LTA test set. In this case, we refer to ECGnet-v1 TL-opt as ECGnet-v1-L, and we consider it as a reference for the comparison. Table 4, instead, reports the main computational characteristics of the three models.

**Table 3:** Performance comparison for the experiment (iii)

Model	Se	Sp	BER	Acc	k	wF1	MF1
ECGnet-v1-L	**92.68 (3.35)**	**99.48 (0.36)**	**3.92 (1.57)**	**98.92 (0.28)**	**92.87 (1.84)**	**98.91 (0.28)**	**96.44 (0.92)**
ECGnet-v1-M	92.49 (2.69)	99.27 (0.21)^†^	4.12 (1.30)	98.71 (0.22)^†^	91.57 (1.47)^†^	98.70 (0.22)^†^	95.78 (0.74)^†^
ECGnet-v1-S	87.93 (5.97)^†^	99.47 (0.54)	6.30 (2.89)^†^	98.50 (0.54)^†^	89.90 (3.60)^†^	98.47 (0.55)^†^	94.94 (1.80)^†^

Performance of three different sizes (L, M, S) of ECGnet-v1, trained with the transfer learning approach and tested on the FT-LTA test set. The best results are marked in bold. Metrics are reported as mean (std) over the 10 models trained with different random initializations.

^†^ Significantly lower (higher in case of BER) than the reference (ECGnet-v1 TL-opt), according to Mann–Whitney U test (*p* < 0.05).

**Table 4:** Computational characteristics comparison for the experiment (iii)

Model	Size	Number of parameters	Inference time
ECGnet-v1-L	39.95 MB	10.5M	~ 1s
ECGnet-v1-M	29.31 MB	7.7M	~ 0.8s
ECGnet-v1-S	**18.67 MB**	**4.9M**	~ **0.6s**

Computational characteristics of the three different sizes (L, M, S) of ECGnet-v1. Inference time refers to the time needed to classify an entire sequence (7 segments) on a CPU (Intel Core i7-10750H). The best values are marked in bold.

As expected, the results reveal an inverse trend between performance and architecture size, according to most of the reported metrics: ECGnet-v1-L is the best performing one, while ECGnet-v1-S is half its size.

Nonetheless, the performance reduction is quite limited for ECGnet-v1-M: sensitivity and BER are not significantly different from the reference ECGnet-v1-L; the other metrics, despite showing a statistically significant decrease, still report very high performance, close to the reference. The deterioration mainly concerns the sensitivity of ECGnet-v1-S, for which we can notice both a lower mean value and a higher standard deviation. The decrease in metrics such as specificity, weighted F1-score, macro F1-score, or accuracy, instead, is fairly narrow, also for ECGnet-v1-S. It is worth noting that both the sensitivity and specificity of ECGnet-v1-M are still above the reference AHA thresholds.

It should also be noted that model size reduction implies inference time shortening from about 1s with ECGnet-v1-L to about 0.6s with ECGnet-v1-S, even if an inference time lower than 1.28s (1 segment duration) is already enough to guarantee real-time prediction without delays.

### Confidence estimation

**Fig. 2:**
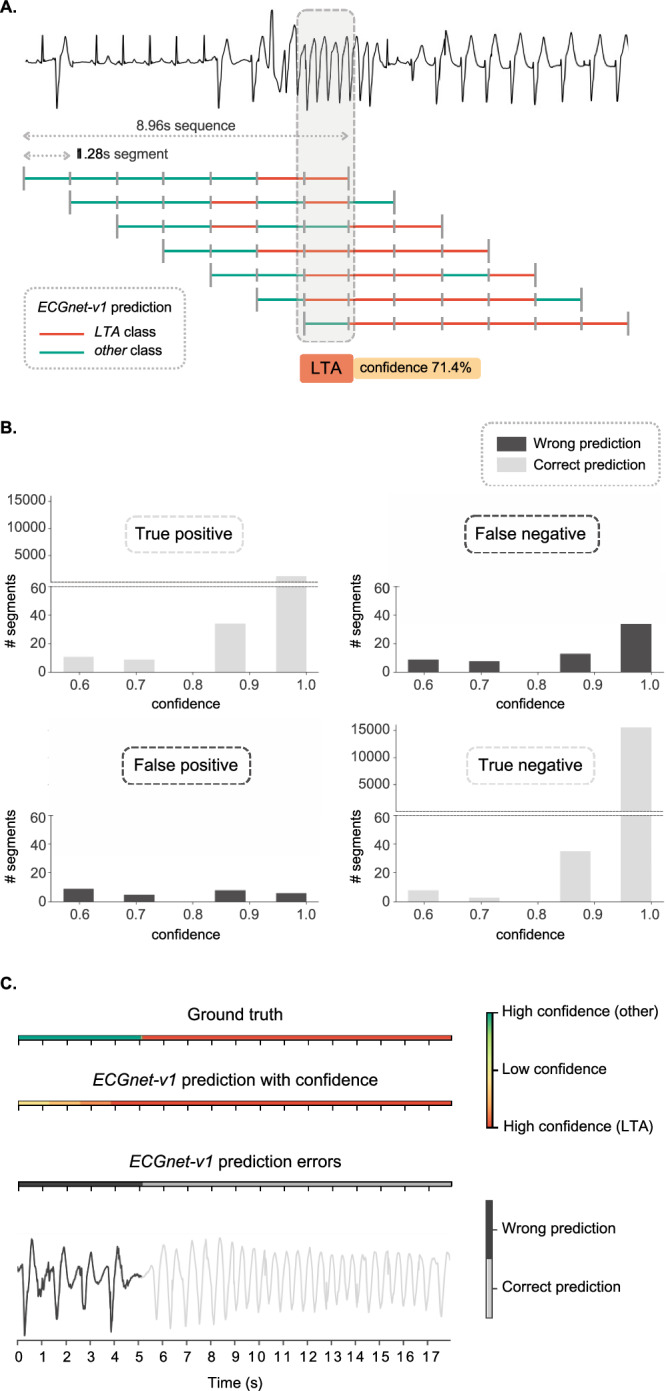
Representation of the confidence estimation mechanism and the related results. **A** Graphical representation of the confidence estimation approach. In this example, the label assigned to the selected segment is LTA and its confidence is 71.4%. **B** Confidence values distribution in the confusion matrix cells. Values are obtained with ECGnet-v1 TL-opt on the FT-LTA test set. **C** Comparison of ground truth, ECGnet-v1 prediction with confidence, wrong/correct prediction, and ECG signal for an excerpt of about 18 seconds of ECG recording. Values are obtained with ECGnet-v1 TL-opt on the FT-LTA test set.

In this section, we propose and test an indicator to estimate the confidence of ECGnet-v1 in detecting LTAs. The details about the confidence computation are reported in paragraph 7 and a graphical summary is provided in Fig. 2A.

Figure 2B shows the distribution of confidence values in the four quadrants (true positive, true negative, false positive, false negative) of the confusion matrix. These results are obtained with the ECGnet-v1 TL-opt model on the FT-LTA test set.

From Fig. 2B, we notice that for correct predictions (true positive and true negative), the confidence distribution is extremely skewed toward higher values. In particular, when confidence is equal to 1, the prediction of the model is correct in almost all cases. For wrong predictions (false positives and false negatives), instead, confidence is more distributed across all the values. The behavior revealed by this graph shows a link between confidence values and prediction errors, suggesting that this measure could be adopted as an indicator of model confidence in prediction.

Based on the intuition that misclassified and low-confidence segments are located in correspondence with transition phases between the two types of rhythm, we compared the performance and confidence of the ECGnet-v1 TL-opt model over the stationary and transitory parts segments of the FT-LTA test set. The aforementioned segments are defined in 7, and the results are reported in Table 5.

**Table 5:** Performance and confidence results of the experiment (iv)

Phase	Se	Sp	BER	Acc	F1	Confidence
Stationary	**96**	**99.86**	**2.07**	**99.57**	**97.09**	**99.84** ± **2.16**
Transitory	59.83	90.43	24.87	75	70.70	94.89 ± 11.76

Performance and confidence of ECGnet-v1, trained with transfer learning approach and tested on two subsets of the FT-LTA test set: stationary-phase segments and transitory-phase segments. The best results are marked in bold.

These results confirm that the model performs considerably better over the stationary phases, compared to the transitory phases. Indeed, the performance is extremely high over the stationary phases, while quite poor over the transitory ones. This is most likely due to the nature of the transition, where the rhythm itself is not well defined, and thus they are harder to define also from a clinical point of view. Further, the confidence value is significantly lower over the transitory phases (*p* < < 0.0001, *r* = 0.196), confirming the previous statement and the link between the confidence and the decrease in performance.

An example of this behavior is shown in Fig. 2C. It reports, for an excerpt of about nine seconds of an ECG recording, the true labels (ground truth), the labels predicted by the model together with its confidence, the segments for which the model provides a wrong prediction, and the ECG signal plot.

Hence, to increase model sensitivity, we should focus on the transition epochs. For instance, the confidence could be exploited to trigger a pre-alert phase in the case of LTAs detected with low confidence. This would imply waiting a few seconds for a more confident prediction to confirm whether to trigger a real alert or not. In case of a false positive, this approach would avoid a waste of resources, while, in case of a false negative, it would reduce the reaction times thanks to the pre-alert trigger. In regions where the Emergency Medical Service (EMS) is overwhelmed, like in high-demand or rural areas, the impact can be noteworthy: reducing false activations saves time for true emergencies while pre-alerting for true alarms enables faster intervention.

## Discussion

In this work, we demonstrate that the exploitation of a transfer learning approach effectively addresses one of the primary limitations in the field of LTA detection: the insufficient availability of ECG recordings containing LTA events. Our results show that the pre-training phase successfully extracts meaningful information from an external dataset. Despite the differences between the pre-training and target dataset and task, the information extracted during pre-training proved to be valuable and transferable to the downstream task. This enabled us to build and leverage a massive dataset by pulling data from another domain (i.e., different types of rhythm and classification tasks), leading to the largest dataset used in this field to date (72’952 recordings from 71’240 different subjects). This is particularly relevant, as a large and diverse pre-training dataset partially compensates for the limited representativeness of the fine-tuning dataset, ultimately enhancing the model’s generalizability. Our model achieves a sensitivity of 92.68% and a specificity of 99.48% on the test data, with a prediction granularity of 1.28 seconds.

The challenge of building a representative training set is critical in both shallow and deep learning approaches. DL- based algorithms possess a greater capacity to learn complex, high-dimensional patterns from big datasets, usually outperforming feature-based approaches. However, this same strength makes the model more susceptible to overfitting. One potential cause is the limited size and the limited size and low representativeness of the training set, which can result in models that struggle to generalize to unseen data, particularly to out-of-domain data. This problem becomes especially critical when attempting to deploy models in real-world applications.

The field of LTALTA detection is particularly vulnerable to these issues, primarily due to the rarity and criticality of these cardiac events, which makes data collection challenging, if not impossible. Additionally, the difficulties associated with data labeling and sharing further compound the problem.

Another innovative aspect of our work is the introduction of an algorithm confidence evaluation mechanism. In the medical field, the impact of false positives and false negatives can be severe. In our specific use-case scenario of automatic alerting of the emergency service in the case of OHCA, the consequences are the delay or lack of rescue in the case of false negatives and a waste of resources in the case of false positives. By integrating a method to evaluate the model’s confidence in its predictions, we add a layer of safety, allowing the system to pre-alert the emergency services in uncertain cases, reducing the impact of misclassifications. Indeed, if the alert is later confirmed, this pre-alert could reduce response time. Conversely, if the alert is canceled, it avoids unnecessary use of resources that could result from a false alarm.

Unlike many previous studies, we do not exclude any data a priori (e.g., intermediate rhythms, transition rhythms, noisy ECGs, or labels) to more closely simulate real-world conditions, even at the cost of potentially reduced performance. Instead of removing segments with transition rhythms, we conducted an additional evaluation by separating stationary rhythms from transition ones. This analysis was enabled by the high prediction granularity of our model (i.e., one prediction for every 1.28-second segment). Our results indicate that the model’s performance was significantly lower during the transition phases, as is always the case when we are trying to discretize a continuous process. The algorithm difficulty reflects the disagreement among cardiologists in labeling data where a transition is present. These results also suggest focusing on transition epochs to further increase model sensitivity. Asking a group of expert cardiologists to more accurately and finely relabel the transition phases, reaching an agreement would be a possible mitigation strategy: the new labels could be used to fine-tune a model with improved performance and enhanced clinical reliability. Moreover, our confidence estimation and pre-alert mechanism can further lessen the consequences of misclassification in the transition phases.

The second major limitation in the literature is the lack of an openly available, well-labeled dataset to be exploited exclusively as an out-of-domain dataset for benchmarking. All the existing studies, including ours, rely solely on testing the algorithms on in-domain datasets, which can result in less realistic performance estimates. This represents a limitation also for our study, which cannot be overcome until a benchmarking dataset is available. Nevertheless, we believe that our algorithm, thanks to the transfer learning approach, can be considered more robust and adaptable by design. Of course, the availability of an out-of-domain benchmarking dataset would provide a more accurate evaluation of model generalizability and allow for a fair comparison of different approaches in the literature. Ideally, such a dataset would be annotated by multiple cardiologists and have a high prediction granularity. The involvement of multiple experts would help mitigate and properly evaluate the impact of ECG segments that are difficult to interpret, while the increased granularity would allow for a more detailed analysis of model performance during rhythm transitions.

Looking ahead, the next step will be the validation and adaptation of our algorithm to data collected with wearable devices, which typically presents greater challenges due to the higher presence of noise and artifacts. To mitigate this, more advanced and customized ECG preprocessing techniques could be applied; moreover, a second step of fine-tuning could be performed to transfer and better adapt the model to the wearable domain. This would be an important step toward ensuring the algorithm’s effectiveness in the real-case scenario. To address potential wearable devices requirements, we also propose lighter models to be able to find the best trade-off between performance and computational requirements, suitable for the specific use case.

Additionally, further studies should investigate the impact of the confidence-based pre-alert approach on overall performance, ideally using a larger test set for a more robust analysis. The future integration of the proposed algorithm into the whole emergency system will also allow for a better evaluation of the practical impact of the confidence-based pre-alert system.

Another promising direction is the exploration of a self-supervised learning approach. This would enable the use of more extensive datasets in the pre-training phase, without labeling-related constraints. This approach could enable the model to learn even more generalized ECG representations, improving adaptability to unseen out-of-domain data. Finally, the big step forward in this field would be the creation of a well-labeled benchmarking dataset, in collaboration with expert cardiologists, to have a common reference openly available to the scientific community.

## Conclusion

In summary, we introduce a robust DL-based model for LTA detection that mitigates the data scarcity problem through transfer learning. Our model extracts knowledge from the biggest dataset employed in similar studies (72’952 ECG recordings) and, after an optimized fine-tuning, effectively addresses the LTA detection task. The results show an improved generalizability and performance. Our model can detect LTAs from single-lead ECGs, with a granularity of 1.28s and with high sensitivity (92.68%) and specificity (99.48%). It also includes a confidence evaluation that, combined with an emergency service pre-alert mechanism, could help to better optimise resources. To conclude, our study effectively addresses the data scarcity issue, advancing LTA detection in wearable monitoring systems and supporting rapid, life-saving interventions in out-of-hospital cardiac arrest emergencies.

## Supplementary information

Supplementary Information

Description of Additional Supplementary files

Supplementary Data 1

Supplementary Data 2

Supplementary Data 3

Reporting Summary

## Data Availability

The ECG data exploited in this study are available in Physionet^17^ with the following identifier(s): “10.13026/34va-7q14”^28^, “10.13026/C2F305”^16^, “10.13026/C22P44”^14^, and “10.13026/C2X59M”^15^. All these data can be accessed directly without any request or application process. The source data to reproduce the plots in Fig. 1 (Supplementary Data 1) and in Fig. 2 (Supplementary Data 2) reported in this manuscript are provided as Supplementary Data. All other data is available from the corresponding author on reasonable request.

## Code availability

The architecture used in this study is based on what was previously developed by Hannun et al.^25^. The code is publicly available on GitHub at https://github.com/awni/ecg^38^.
