# Supplementary material for: Overcoming data scarcity in life-threatening arrhythmia detection through transfer learning
Source: Commun Med (Lond). 2025 Jul 1;5:248. doi: 10.1038/s43856-025-00982-9 (PMC12215667; doi:10.1038/s43856-025-00982-9)
Supplement: Supplementary file 1 — Supplementary Information [file 43856_2025_982_MOESM1_ESM.pdf]

Overcoming data scarcity in life-threatening arrhythmia detection through transfer learning

Giuliana Monachino[1,2*], Beatrice Zanchi[1,3], Michael Wand[4,1], Giulio Conte[5,6], Athina Tzovara[2,7], Francesca Dalia Faraci[1]

[1*]Institute of Digital Technologies for Personalized Healthcare - MeDiTech, Department of Innovative Technologies, University of Applied Sciences and Arts of Southern Switzerland, Via la Santa 1, Lugano, 6900, Switzerland.
[2]Institute of Informatics, University of Bern, Neubrückstrasse 10, Bern, 3012, Switzerland.
[3]Department of Quantitative Biomedicine, University of Zurich, Schmelzbergstrasse 26, Zurich, 8091, Switzerland.
[4]Dalle Molle Institute for Artificial Intelligence, USI-SUPSI, Via la Santa 1, Lugano, 6900, Switzerland.
[5]Cardiology Department, Cardiocentro Ticino Institute, Ente Ospedaliero Cantonale, Via Tesserete 48, Lugano, 6900, Switzerland.
[6]Faculty of Biomedical Sciences, Università della Svizzera Italiana, Via la Santa 1, Lugano, 6900, Switzerland.
[7]Sleep Wake Epilepsy Center | NeuroTec, Department of Neurology, Inselspital, Bern University Hospital, University of Bern, Freiburgstrasse 16, Bern, 3010, Switzerland.

*Corresponding author(s). E-mail(s): giuliana.monachino@supsi.ch;

SUPPLEMENTARY INFORMATION

Table 1: Characteristics of PT dataset and its source datasets

	Train			Validation			Test			Total		
	N sbj	N rec	N seq	N sbj	N rec	N seq	N sbj	N rec	N seq	N sbj	N rec	N seq
Chapman	2885	2885	2885	2598	2598	2598	4691	4691	4691	10174	10174	10174
Georgia	1813	1813	1813	1272	1272	1272	2544	2544	2544	5629	5629	5629
Ningbo	22072	22072	22072	5247	5247	5247	5995	5995	5995	33314	33314	33314
PTB-XL	16374	18727	18727	1665	1947	1947	240	288	288	18279	20962	20962
Tot	43144	45497	45497	10782	11064	11064	13470	13518	13518	67396	70079	70079

Number of subjects (N sub), recordings (N rec), and 7-segment sequences (N seq) in each source dataset for each subset (train, validation, and test) in the PT dataset.

Table 2: Labels distribution in PT dataset

	Train		Validation		Test		Total	
	N seq	N segm	N seq	N segm	N seq	N segm	N seq	N segm
N	17441	122087	4176	29232	5951	41657	27568	192976
SB	11226	78582	2797	19579	3536	24752	17559	122913
ST	5696	39872	1417	9919	2057	14399	9170	64190
AFL	4754	33278	1186	8302	1527	10689	7467	52269
AF	2488	17416	552	3864	2036	14252	5076	35532
SA	1472	10304	356	2492	452	3164	2280	15960
SA, SB	510	3570	127	889	159	1113	796	5572
P	450	3150	102	714	123	861	675	4725
SVT	436	3052	108	756	140	980	684	4788
N, SA	410	2870	96	672	123	861	629	4403
N, SB	234	1638	55	385	65	455	354	2478
N, ST	156	1092	36	252	44	308	236	1652
ARH	129	903	32	224	40	280	201	1407
AT	95	665	24	168	36	252	155	1085
Tot	45497	318479	11064	77448	16289	114023	72850	509950

Number of 7-segment sequences (N seq) and 256-sample segments (N segm) in each rhythm class (or group of classes in case of multiple labels) for each subset (train, validation, and test) in PT dataset. N: normal sinus rhythm, SB: sinus bradycardia, ST: sinus tachycardia, AFL: atrial flutter, AF: atrial fibrillation, SA: sinus arrhythmia, P: paced rhythm, SVT: supraventricular tachycardia, ARH: atrial rhythm, AT: atrial tachycardia

Table 3: Characteristics of FT-LTA dataset and its source datasets

	Train			Validation			Test			Total		
	N sbj	N rec	N seq	N sbj	N rec	N seq	N sbj	N rec	N seq	N sbj	N rec	N seq
CUDB	23	23	915	5	5	145	7	7	324	35	35	1384
MITDB	29	29	3549	7	7	623	9	9	1279	45	45	5451
VFDB	15	15	2950	3	3	406	4	4	908	22	22	4264
Tot	67	67	7414	15	15	1174	20	20	2511	102	102	11099

Number of subjects (N sub), recordings (N rec), and 7-segment sequences (N seq) in each source dataset for each subset (train, validation, and test) in the FT-LTA dataset.

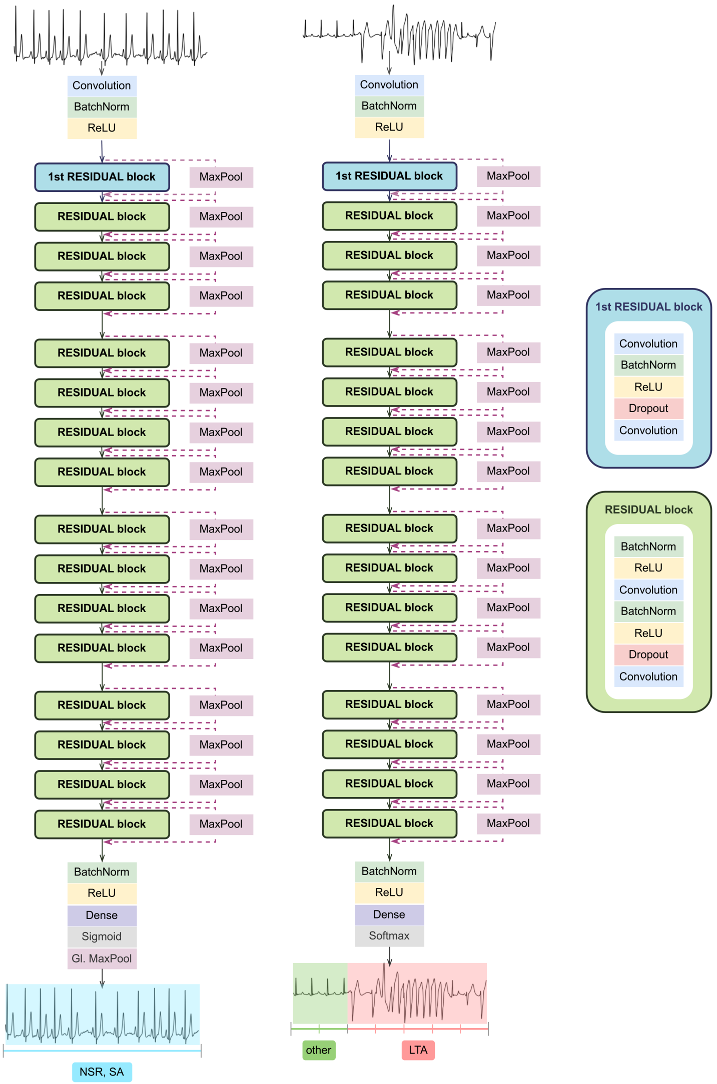

Fig. 1: **ECGnet-v0 and ECGnet-v1 architecture.** Architecture scheme of ECGnet-v0 and ECGnet-v1, showing the common structure of stacked residual blocks and their composition, and the different final classification heads.

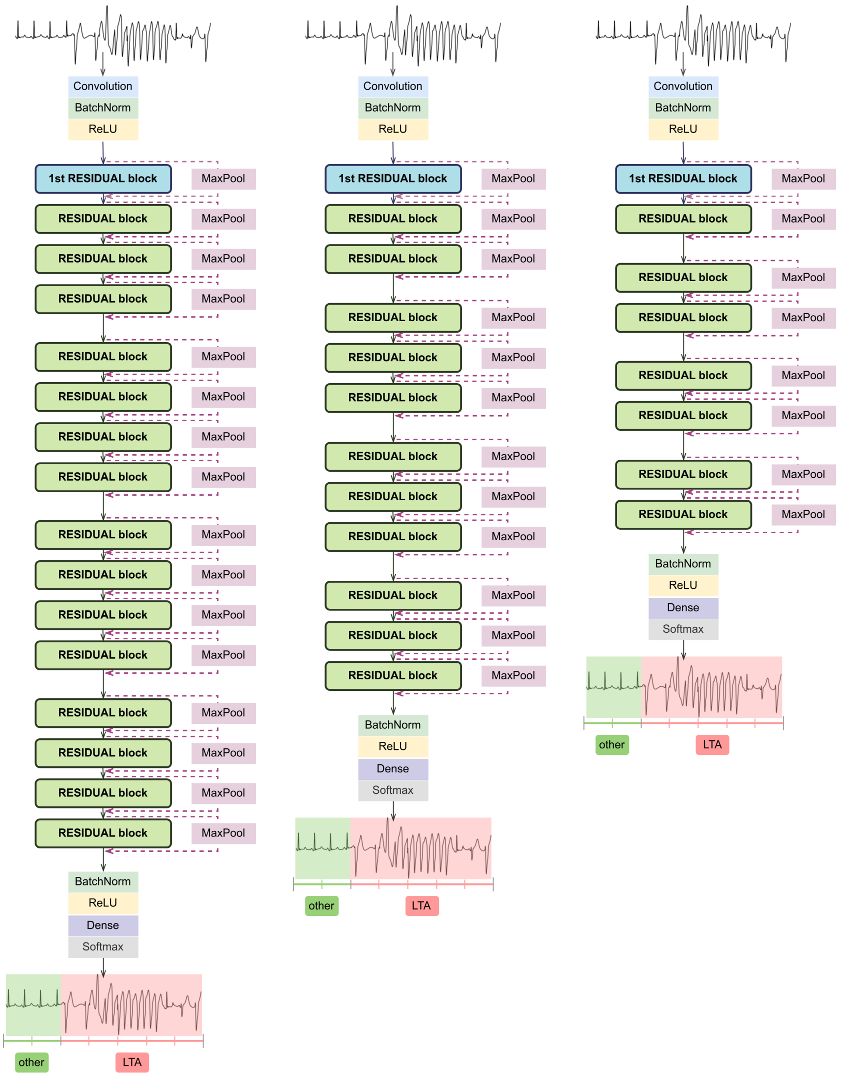

Fig. 2: **ECGnet-v1-L, ECGnet-v1-M and ECGnet-v1-S architecure.** Architecture scheme of ECGnet-v1-L, ECGnet-v1-M, and ECGnet-v1-S, showing the different number of stacked residual blocks, and the common final classification heads.

Table 4: Performance comparison for the experiments (i) and (ii) without batch balancing

Model	Se	Sp	BER	Acc	k	wF1	MF1
ECGnet-v1 TS	87.85 (3.58)	99.05 (0.47)	6.55 (1.82)	98.12 (0.53)	87.64 (3.37)	98.11 (0.52)	93.82 (1.68)
ECGnet-v1 TL	85.67 (10.19)	99.31 (0.56)	7.51 (4.97)	98.17 (0.77)	87.43 (6.42)	98.12 (0.88)	93.71 (3.23)
ECGnet-v1 TL opt	**90.47 (4.32)** *	**99.54 (0.26)** *	**4.99 (2.07)** *	**98.78 (0.27)** *	**91.86 (1.96)** *	**98.77 (0.28)** *	**95.93 (0.98)** *

Performance of ECGnet-v1 TS (training from scratch), ECGnet-v1 TL (transfer learning), and ECGnet-v1 TL-opt (transfer learning with optimized configuration) computed on the FT-LTA test set. The models have been trained without batch balancing. Metrics are reported as mean (std) over the 10 models trained with different random initializations. The best results are marked in bold.

*Significantly higher (lower in case of BER) than the baseline (ECGnet-v1 TS), according to Mann-Whitney-U-test ($p < 0.05$).

Table 5: Performance comparison for the experiments (i) and (ii) with weighted binary cross-entropy loss

Model	Se	Sp	BER	Acc	k	wF1	MF1
ECGnet-v1 TS	86.61 (7.54)	98.95 (0.82)	7.22 (3.53)	97.92 (0.61)	86.28 (3.81)	97.91 (0.59)	93.13 (1.91)
ECGnet-v1 TL	91.25 (6.23) *	98.68 (0.68)	5.04 (2.89) *	98.06 (0.45)	87.64 (2.87)	98.08 (0.44)	93.82 (1.43)
ECGnet-v1 TL opt	**93.01 (2.05)** *	**99.23 (0.27)**	**3.88 (0.95)** *	**98.71 (0.19)** *	**91.64 (1.17)** *	**98.72 (0.18)** *	**95.82 (0.59)** *

Performance of ECGnet-v1 TS (training from scratch), ECGnet-v1 TL (transfer learning), and ECGnet-v1 TL-opt (transfer learning with optimized configuration) computed on the FT-LTA test set. The models have been trained without batch balancing and exploiting a weighted binary cross-entropy loss. Metrics are reported as mean (std) over the 10 models trained with different random initializations. The best results are marked in bold.

*Significantly higher (lower in case of BER) than the baseline (ECGnet-v1 TS), according to Mann-Whitney-U-test ($p < 0.05$).

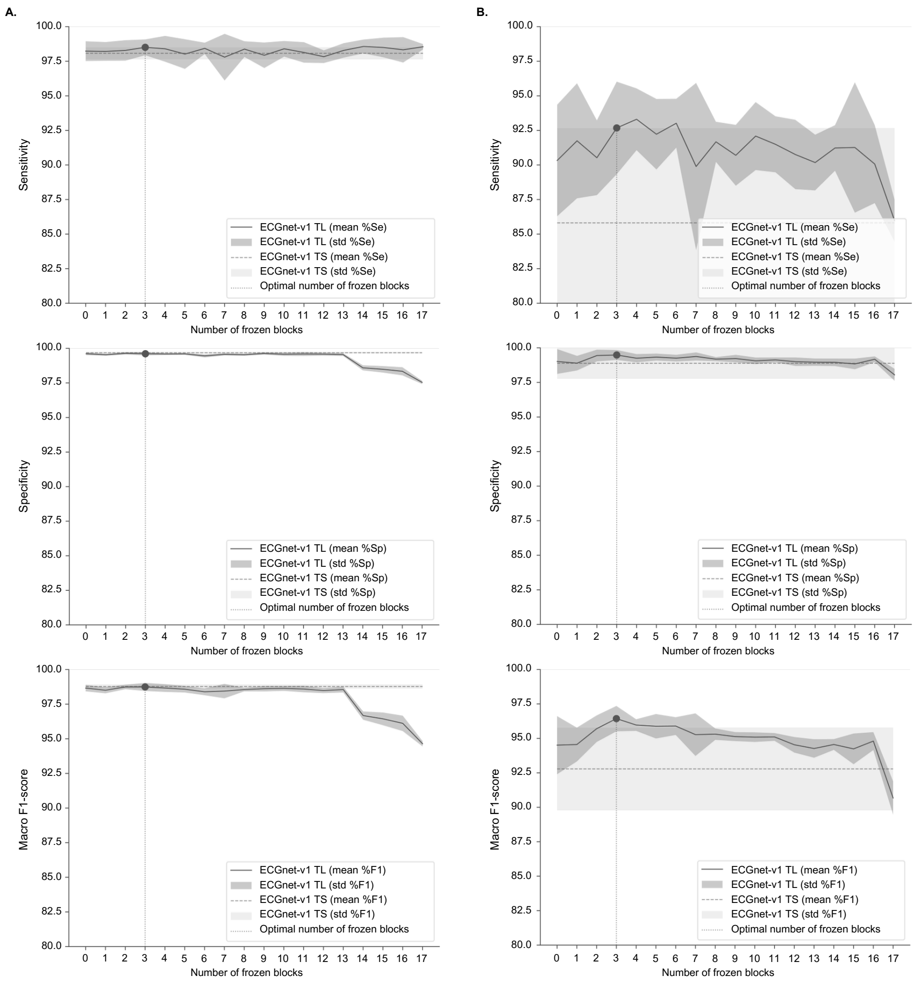

Fig. 3: **Sensitivity, specificity, and macro F1-score of ECGnet-v1 TL on the FT-LTA validation and test set, varying the freezing configuration.** a) Validation set performance. b) Test set performance. The x-axis reports the number of frozen blocks during the fine-tuning phase (from no frozen blocks to all but the last frozen block). The lines and the shadows represent, respectively, the means and the standard deviations across the n=10 runs.
