# Supplementary material for: Overcoming data scarcity in life-threatening arrhythmia detection through transfer learning
Source: Commun Med (Lond). 2025 Jul 1;5:248. doi: 10.1038/s43856-025-00982-9 (PMC12215667; doi:10.1038/s43856-025-00982-9)
Supplement: Supplementary file 2 — Description of Additional Supplementary files [file 43856_2025_982_MOESM2_ESM.pdf]

Description of Additional Supplementary files

File name: Supplementary Data 1

Description: Source data for the sensitivity, specificity, and macro-F1 score validation comparative plots reported in Figure 1 and in Supplementary Figure 3a.

File name: Supplementary Data 2

Description: Source data for the histogram reported in Figure 2b.

File name: Supplementary Data 3

Description: Source data for the sensitivity, specificity, and macro-F1 score test comparative plots reported in Supplementary Figure 3b.
